# Diurnal Cortisol Secretion Is Not Related to Multiple Sclerosis-Related Fatigue

**DOI:** 10.3389/fneur.2019.01363

**Published:** 2020-01-28

**Authors:** Arjan Malekzadeh, Ilona Bader, Julia van Dieteren, Annemieke C. Heijboer, Heleen Beckerman, Jos W. R. Twisk, Vincent de Groot, Charlotte E. Teunissen

**Affiliations:** ^1^Neurochemistry Laboratory, Department of Clinical Chemistry, VU University Medical Center, Amsterdam, Netherlands; ^2^Endocrinology Laboratory, Department of Clinical Chemistry, VU University Medical Center, Amsterdam, Netherlands; ^3^Department of Rehabilitation Medicine, VU University Medical Center, Amsterdam, Netherlands; ^4^MS Center Amsterdam, VU University Medical Center, Amsterdam, Netherlands; ^5^Amsterdam Public Health Research Institute, VU University Medical Center, Amsterdam, Netherlands; ^6^Department of Clinical Epidemiology and Biostatistics, VU University Medical Center, Amsterdam, Netherlands

**Keywords:** multiple sclerosis, fatigue, cognitive behavioral therapy, exercise therapy, energy conservation management, hypothalamus–pituitary adrenal axis, diurnal cortisol

## Abstract

Some evidence supports the involvement of the hypothalamic–pituitary–adrenal axis (HPA axis) with multiple sclerosis (MS)-related fatigue. In this study, we determined the relation of HPA-axis function with primary fatigue in MS patients in the longitudinal treating fatigue in a MS cohort. MS patients from the TREeating FAtigue in MS (TREFAMS) research program that consists of three randomized controlled trials to study the effects of aerobic training, energy conservation management, and cognitive behavioral therapy on MS-related fatigue were included. The HPA-axis functioning was determined at baseline, the end of treatment (16 weeks) and after 52 weeks. The cortisol awakening response (CAR) and night-time cortisol levels were analyzed. Fatigue was measured with the fatigue subscale of the Checklist Individual Strength (CIS20r fatigue). There was no relationship between CAR and night-time cortisol parameters with CIS20r fatigue scores. Neither of the treatments influenced CAR and night-time cortisol parameters, with the exception of an effect in the energy conservation management treatment group on the CAR surge increase over 52 weeks (β = −114.8, *p* = 0.007, 95% CI = −197.6, −31.9). Our data suggest that the diurnal cortisol secretion is not associated with MS-related fatigue. This indicates that MS-related fatigue is not attributed to diurnal cortisol secretion and is likely caused by other disease mechanisms.

## Introduction

Fatigue is the most commonly reported symptom in multiple sclerosis (MS), affecting ~80% of MS patients (1–4). MS-related fatigue is considered to be one of the main causes of impaired quality of life and is often considered to be the most debilitating symptom (4). MS-related fatigue negatively affects social participation and can lead to socioeconomic problems (5). Fatigue in MS remains poorly understood and is often underemphasized because of its subjective nature and lack of consensus on the definition of fatigue. Fatigue can be defined as an “overwhelming, debilitating, and sustained sense of exhaustion that decreases one's ability to carry out daily activities, including the ability to work effectively and function at one's usual level in family or social roles” (6). The different fatigue definitions and domains indicate that fatigue is considered to be a multifaceted symptom.

The exact pathophysiological mechanism behind MS-related fatigue is currently unknown. Most likely MS-related fatigue is multifactorial, and various pathophysiological mechanisms have been proposed: (1) dysregulation of the immune system, (2) dysfunction of the central nervous system caused by lesion formation, (3) impaired nerve conduction, (4) energy depletion, (5) involvement of the autonomic nervous system, (6) neurotransmitter dysregulation, and (7) dysregulation of hypothalamic–pituitary–adrenal axis (HPA axis) (3, 6–9). A hyperactivity of the HPA axis in MS patients with fatigue in comparison to MS patients without fatigue was observed in a cross-sectional studies (7, 10). The HPA axis regulates the diurnal cortisol secretion, and upon awakening, a surge of in cortisol levels is observed, known as the cortisol awakening response (CAR) (11). Cortisol secretion can be measured in different body fluids, such as saliva, blood, and urine (11). Cortisol levels decrease during the day, with lowest concentrations at night (11). To test whether the HPA-axis feedback mechanisms work properly, often a dexamethasone suppression test (DST) is performed (11). Intake of low-dose dexamethasone before sleeping initiates a negative feedback of the HPA-axis cortisol secretion, which leads to decreased CAR upon awakening (11). Non-suppression after dexamethasone during a relapse in relapse remitting patients has been shown and could attribute to the observed hyperactive CAR in MS patients (12). It is possible that the dysregulation of the HPA axis could be involved in MS-related fatigue. However, results have been inconsistent so far (7).

Accumulating evidence supports the effectiveness of non-pharmacological rehabilitation therapies such as aerobic training (AT), energy conservation management (ECM), and cognitive behavioral therapy (CBT) for alleviating MS-related fatigue (13–15). However, only limited number of randomized controlled trials have focused on MS-related fatigue as primary outcome measure. The TREeating FAtigue in MS (TREFAMS) program was focused on MS-related fatigue as primary outcome measure (13–16). Overall, in all three different intervention groups, a similar pattern of Checklist of Individual Strength (CIS20R) fatigue scores was observed, in which a mean decline of CIS20R fatigue scores for the intervention groups was visible after the initial 16 weeks of therapy (13–15). Only for the CBT intervention group, a significant reduction of −6.7 (95% CI = −10.7; −2.7) of CIS20R fatigue scores was observed compared to the control group, which diminished post-intervention (52 weeks) (13).

Based on the earlier observations of hyperreactivity of the HPA axis in MS-related fatigue and the interesting results observed for the rehabilitation treatments, we hypothesized that rehabilitation treatments AT, CBT, or and ECM are able to reduce MS-related fatigue due to normalization of the HPA axis (7, 17, 18). Therefore, the primary aim of this study was to determine the longitudinal effect of HPA-axis function on MS-related fatigue, by assessing diurnal cortisol saliva levels in patients that participated in the TREFAMS research program that consisted of three randomized controlled trials to study the effects of AT, ECM, and CBT (17–19). Moreover, we investigated whether specific treatments affect diurnal cortisol saliva secretion.

## Methods

### Study Design

This study is a part of the TREFAMS-ACE research program (16). Briefly, the TREFAMS-ACE program is a multicenter program that includes three single-blinded randomized controlled trials (RCTs) with repeated measurements in time (0, 8, 16, 26, and 52 weeks). In this study, the effectiveness of rehabilitation treatments AT, ECM, and CBT on reducing MS-related fatigue, with fatigue as primary outcome was determined. Each RCT applied a two-parallel-arm design with (1) an intervention group (2) and a control group. The intervention consisted of 12 therapist-led sessions in 16 weeks, and the control group received three consultations in 4 months given by an experienced MS nurse (16).

The inclusion criteria for the TREFAMS-ACE program consisted of (a) definitive MS diagnosis, (b) 1 week prior inclusion fatigue scores CIS20R ≥ 35, (c) ambulatory patients, (d) no signs of exacerbation or corticosteroid treatment in the past 3 months, (e) no current infections (normal leukocyte and CRP counts), (f) no anemia (normal hemoglobin and hematocrit in blood), and (g) normal thyroid (normal thyroid-stimulating hormone levels in blood). The exclusion criteria were (a) depression (Hospital Anxiety and Depression Scale depression >11), (b) primary sleep disorders, (c) severe comorbidity (Cumulative Illness Rating Scale item score ≥3), (d) current pregnancy or given birth in the past 3 months, and (e) non-pharmacological and pharmacological treatments for fatigue started within the last 3 months (16). The medical ethics committee of the VU University Medical Center approved the TREFAMS-ACE program, and local feasibility statements were obtained from each participating medical center (16). This study was funded by Fonds NutsOhra (ZonMw 89000005). The three RCTs were registered in advance (ISRCTN 69520623, ISRCTN 58583714, and ISRCTN 82353628).

### Study Participants

**Figure 1:**
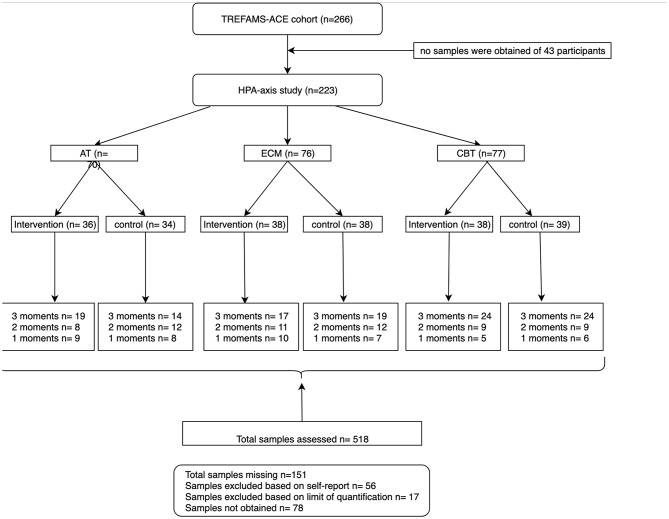
Flow chart of participants included from the TREFAMS-ACE cohort with different measurement moments.

For this HPA-axis study, we included 223 of the 266 patients included in the TREFAMS cohort (Figure 1). We obtained CAR values of 117 participants with all the three measurement moments (0, 16, and 52 weeks), 61 patients with CAR values for two different measurement moments, and 45 patients with CAR values for one measurement moments. The linear mixed model (LMM) analyses of the AT trial group were based on 19 patients with all the three measurement moments, eight patients with two different measurement moments, and nine with one measurement moments. The ECM group consisted of 17 participants with three different measurement moments, 11 with two measurement moments, and 10 with one measurement moments. For the CBT group, valid CAR values were obtained from 24 participants with three measurement moments, nine with two measurement moments, and five with one. The pooled control group consisted of 57 participants with three measurement moments, 33 with two measurement moments, and 21 with one measurement moments.

Of 43 participants, no saliva samples were obtained on the assessed measurement moments and were therefore excluded from start. Moreover, based on self-reports, a total of 56 samples from different timepoints were excluded from further analyses because delayed CAR collection, eating, or drinking before collection were reported. Moreover, we were not able to quantify the CAR for another 17 samples at one or more collection timepoints due to the fact that CAR levels were below the limit of quantification. Lastly, 78 samples at different measurements were not collected (Figure 1).

### Fatigue Measurements and Scores

Fatigue was measured with the CIS20R, subscale subjective fatigue. The CIS20R is a multidimensional questionnaire and consists of 20 items on a 7-point scale (20). These 20 items are divided into four subscales: (1) subjective fatigue, (2) concentration, (3) motivation (4), and activity. The fatigue score varies between 8 and 56 points. To define the severity of fatigue a cutoff of 35 (CIS20R, subscale fatigue 35) was applied (16, 21). CIS20R fatigue scores were collected for all the measurement moments (0, 16, and 52 weeks).

### Salivary Cortisol Sampling and Dexamethasone Suppression Test

HPA-axis functioning of each patient was assessed by analyzing cortisol levels in saliva throughout the day at measurement moments 0, 16 (at the end of the intervention), and 52 weeks after randomization. Saliva was collected at home, 5 days' post-planned sessions; upon awakening timepoint 1 (T1), 30 (T2), 45 (T3), and 60 (T4) min after awakening in saliva tubes with cotton swabs (Sarstedt, Germany). A fifth sample was collected at 2,200 h (T5). Moreover, 0.5 mg dexamethasone was ingested before sleeping, and a sixth sample of saliva was collected the next morning upon awakening (T6). A DST was done to assess the negative feedback mechanisms of the HPA axis. The participants were instructed not to smoke, eat, drink, or brush teeth within 15 min before saliva collection. Moreover, participants were instructed to report other potential CAR-interfering factors (e.g., sleeplessness before the sampling day, having the flue or flu-like symptoms) on the provided forms. Samples with reported potential interfering factors were excluded for further analyses. Samples were stored at refrigerators, and participants were instructed to return the collected samples by mail upon collection of T6. Upon arrival, the collected saliva was stored at −20°C.

### Salivary Cortisol Quantification

Before the measurements, the saliva samples were thawed for ~1 h at room temperature. Subsequently, the tubes were centrifuged at 2,000 × *g* for 10 min, the cotton swabs were discarded, and the residual saliva was stored at 4°C or directly used.

Cortisol levels were determined using supported liquid extraction+ (SLE+) plates (Biotage, Sweden) and liquid chromatography with tandem mass spectrometry (LC-MS/MS). First, all tubes were vortexed and centrifuged at 1,900 × g for 5 min at 15°C. For calibration curve, a 73 times diluted cortisol standard (C-106) (Cerilliant, TX, USA) was serially diluted in deionized water (5 × dilution). In addition, three internal cortisol controls (high, middle, and low) (C-106) diluted in artificial saliva (Saliva Orthana) were included to assess assay performance. Moreover, an isotope-labeled internal standard, ^13^C_3_-cortisol (IsoSciences, PA, USA), was diluted 22.2 times in deionized water, and 100 μl was added to each well of a Nunc™ 96-Well Polypropylene MicroWell™ Plate (Thermo Scientific, MA, USA), already containing 100 μl of diluted work standard, internal controls, or saliva samples. Next, the Nunc™ 96-Well Polypropylene MicroWell™ Plate was vortexed for 15 min at room temperature, and the content was pipetted in the SLE+ plate 200 μl (Biotage, Uppsala, Sweden). One milliliter methyl-tert-butylether (Biosolve, The Netherlands) was added in each column of the SLE+ plate to elute the cortisol. An Axygen 96-well plate (Axygen Scientific, CA, USA) was used to capture the eluate. Subsequently, this solution was evaporated with a nitrogen sample concentrator (Techne, NJ, USA). The residue was dissolved in 150 μl 50% methanol and centrifuged for 5 min at 1,900 × *g*. Finally, LC-MS/MS analysis was conducted using the Acquity UPLCS H-class System (Waters, MA, USA) coupled to a Quattro Premier XE™ Tandem Mass Spectrometer (Waters, MA, USA) with Masslynx™ v4.1 software. A Synergy Hydro RP column (100 × 2 mm, 4 μm, Phenomenex, CA, USA) protected by the Securityguard C18 guard column (4 × 2 mm, Phenomenex, CA, USA) was used for separation of analytes. After detection of cortisol, the TargetLynx method was used to calculate the cortisol concentrations. Using this method, peaks are integrated, the calibration curve is calculated, and finally, sample cortisol concentrations were calculated in nanomoles per liter. The chromatograms were checked and, if necessary, manually adjusted. The dynamic range for cortisol quantification was between 0.5 and 600 nmol/L. Intra-assay variability coefficient of variation (CV%) for concentrations lower than 1 nmol/L was <18%, whereas the intra-assay CV% for cortisol concentrations was higher than 1 nmol/L was <7%.

### Saliva Cortisol Calculations

To get insight into diurnal cortisol secretion in saliva, we determined the area under the curve with respect to ground (AUCg) and increase (AUCi), and night time cortisol, and HPA-axis feedback mechanisms were assessed by including a DST. The AUCg is an estimate of the total cortisol secretion and predicts the mean cortisol level throughout the day, whereas the AUCi is a measure of the dynamic changes of the CAR and is more sensitive to emphasize changes over time (19, 22). The AUCg and AUCi were calculated using the cortisol values (nmol/L) of T1–T4 saliva samples (1-h post-awakening). We only included and calculated AUC of measurement moments when all timepoints post-awakening (T1–T4) were determined. Both AUCg and AUCi were calculated as described earlier (19). Night-time cortisol assessment was a single assessment of T5 saliva sample. To identify the suppressors and non-suppressors on the DST, we divided the cortisol levels of T1 by cortisol levels at T6. For the DST, different inhibition ranges are often applied varying between 2.8 and 4 nmol/L as suppressor cutoffs (21, 23). The cutoff we applied for dexamethasone suppressors is 4.0 nmol/L or a higher T1/T6 ratio >2.4 (based on mean T1 levels divided by the 4 nmol cutoff). A total of 16 different samples of different timepoints were excluded from the DST analyses based on self-reports, in which participants reported to not have taken the dexamethasone pill.

### General Statistical Analysis

Non-parametric statistics (Mann–Whitney or Kruskal–Wallis) were applied to assess baseline values between treatment groups. To assess baseline correlations, Spearman Rho test was applied. Based on the nested nature of the longitudinal data, LMM analyses was used to analyze the data of the combined cohort. For all the LMM analyses, a random intercept and corrections for respective baseline values of the dependent variable were performed. All patients (*n* = 223) with at least one valid AUCi, AUCg, night-time cortisol, and DST scores were included because LMM analyses can adequately interpolate missing values. All analyses were carried out in SPSS 23.0. A significance threshold of *p* < 0.05 was set.

#### LMM Analyses of Diurnal Cortisol Parameters on CIS20r Fatigue in TREFAMS-ACE Cohort

To assess the longitudinal association of the diurnal cortisol parameters with MS-related fatigue, LMM analyses with the four diurnal cortisol parameters (AUCg, AUCi, night-time cortisol, and DST) as independent variables and the CIS20R subscale fatigue as continuous dependent variables were performed. The model was corrected for baseline CIS20R fatigue scores. Diurnal cortisol parameters were separately included as covariates. Potential confounding or effect modification by age, gender, disease duration, and Expanded Disability Status Scale (EDSS) was assessed for both models. Confounding was considered present if the coefficient of the independent cortisol variable changed by more than 10% after entering the confounder. The confounder was then retained in the model. Effect modification was considered present when the interaction term cortisol parameter × confounding variable was significant.

#### The Effect of Different Interventions on Diurnal Cortisol Parameters

LMM analyses was also used to determine the effects of AT, CBT, and ECM on the four diurnal cortisol parameters (AUCg, AUCi, S5, and DST). Four different models with a diurnal cortisol parameter as dependent outcome variable and treatment as independent variable were constructed. The effect of the same potential confounders and effect modifiers were assessed.

## Results

### Participants

Data of 223 participants were included in the analyses (Figure 1). No significant differences were observed in baseline characteristics between the three intervention groups and the pooled control group, for age, disease duration, EDSS, AUCg, AUCi, and CIS20R fatigue scores (Table 1). Furthermore, no correlations were observed between the diurnal cortisol parameters with CIS20R fatigue scores and EDSS at baseline. In the total group, female participants had higher baseline AUCg (Mann–Whitney *U* = 14,138, *Z* = −3.44, *p* = 0.001) and AUCi (Mann–Whitney *U* = 14,193, *Z* = −3.39, *p* = 0.001) than male participants. Male participants were significantly older (Mann–Whitney *U* = 11,502, *Z* = −2.86, *p* = 0.004) and had higher EDSS scores at baseline (Mann–Whitney *U* = 8,560, *Z* = −3.12, *p* = 0.002). At baseline, the DST showed an overall non-suppression of 6% in the total group of participants, indicating proper negative feedback mechanisms of the HPA axis in 94% of the participants. Lastly, mean baseline characteristics age, disease duration, gender, and EDSS values of the excluded participants (*n* = 43) did not differ with the included participants.

**Table 1:** Characteristics of the participants.

	**AT** **(*n* = 36)**	**ECM (*n* = 38)**	**CBT (*n* = 38)**	**Controls (*n* = 111)**
**Patient characteristics**				
Male	9	7	12	28
Female	27	31	26	83
Age (baseline, mean, SD) (years)	43.6 (11.3)	47.9 (11.4)	50.8 (8.6)	48.0 (9.6)
Disease duration (baseline, mean, SD) (years)	6.6 (5.3)	9.8 (8.6)	8.7 (7.7)	10.5 (7.7)
EDSS (baseline) (mean, SD)	2.6 (1.2)	2.8 (1.6)	2.6 (1.6)	2.7 (1.5)
**Type of MS**				
Relapsing remitting	25	29	26	81
Primary progressive	4	2	6	9
Secondary progressive	4	7	5	18
Unknown/other	3		1	3
**CIS20r subscale fatigue, mean (SD)**				
0 weeks	41.7 (7.8)	43.5 (8.8)	42.3 (8.5)	42.7 (7.4)
16 weeks	36.7 (8.9)	39.1 (8.7)	31.0 (10.7)	41.9 (8.2)
52 weeks	43.1 (6.8)	41.4 (8.7)	37.8 (10.1)	39.9 (10.2)
**AUCg, mean (SD) (nmol/L/h)**				
0 weeks	805 (311)	735 (333)	750 (310)	765 (326)
16 weeks	854 (394)	656 (328)	790 (330)	784 (341)
52 weeks	855 (502)	707 (292)	820 (490)	721 (357)
**AUCi, mean (SD) (nmol/L/h)**				
0 weeks	249 (341)	215 (354)	150 (380)	137 (327)
16 weeks	250 (346)	121 (282)	110 (290)	245 (359)
52 weeks	201 (448)	115 (422)	230 (360)	225 (328)
**S5, night time cortisol mean (SD) (nmol/L)**				
0 weeks	1.0 (0.8)	2.2 (4.6)	2.4 (4.6)	1.5 (1.7)
16 weeks	1.0 (1.0)	1.2 (1.0)	1.0 (0.6)	1.6 (2.4)
52 weeks	2.0 (2.5)	1.1 (0.6)	1.0 (0.8)	1.4 (1.2)
**DST ratio mean (SD)**				
0 weeks	16.9 (13.6)	12.3 (6.7)	18.9 (21.5)	16.6 (11.6)
16 weeks	15.1 (7.9)	12.6 (8.9)	15.9 (7.3)	13.5 (10.3)
52 weeks	16.2 (11.4)	15.7 (15.9)	16.5 (11.2)	12.9 (9.4)

*EDSS, expanded disability status scale; CIS20r, checklist individual strength 20r; AUCg, area under the curve with respect to ground; AUCi, area under the curve with respect to increase; DST, dexamethasone suppression test; AT, aerobic training; ECM, energy conservation management; CBT, cognitive behavioral therapy*.

### Effectiveness of Treatments to Reduce Fatigue

**Figure 2:**
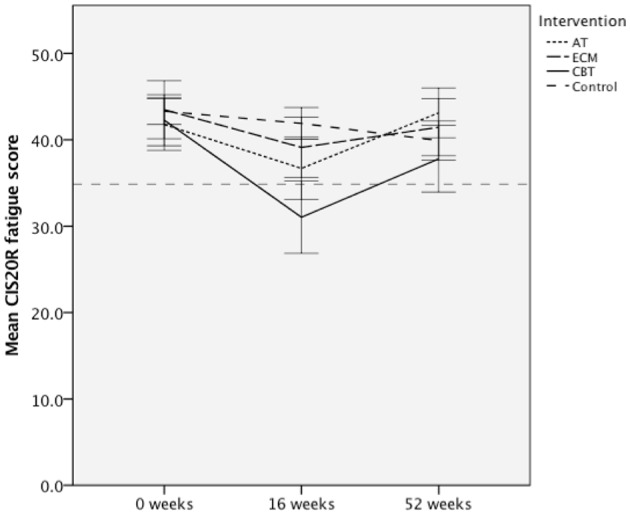
Mean Checklist individual Strength 20R (CIS20R) fatigue scores for the aerobic training (AT), energy conservation management (ECM), cognitive behavioral therapy (CBT) intervention groups, and pooled control for each assessed timepoint. Dotted line represents a CIS20r subscale fatigue cutoff of 34. Error bars represent 95% confidence interval (CI).

The overall goal of the TREFAMS-ACE study was to determine whether AT, ECM, and CBT rehabilitation interventions are able to reduce MS-related fatigue (CIS20R <35) and improve social participation in MS patients (13–16). Overall, in all three different intervention groups, a similar pattern of CIS20R fatigue scores was observed, in which a mean decline of CIS20 fatigue scores for the intervention groups was visible after the initial 16 weeks of therapy, especially for the AT and CBT intervention groups (Figure 2). This effect diminished post-intervention (16–52 weeks) (Figure 2). Only a significant estimated reduction of 2.77 (*p* = 0.003, 95% CI = −4.61, −0.94) in the CIS20r fatigue score was observed for the CBT intervention group over 52 weeks (Figure 2). Further assessment of the CBT intervention group shows a stronger effect during the intervention period (0–16 weeks), with an estimated reduction of CIS20r fatigue score of 4.0 (*p* < 0.001, 95% CI = −5.86, −2.14).

### The Longitudinal Effect of Diurnal Cortisol Secretion on MS-Related Fatigue

No significant relationships were found between longitudinal changes in AUCg, AUCi, night-time cortisol levels, or DST ratios and longitudinal changes in CIS20R fatigue scores. Overall, no confounding and effect-modifying effects of age, gender, disease duration, or baseline EDSS were observed (Table 2).

**Table 2:** Linear mixed model results for the effects of diurnal cortisol secretion on CIS20r fatigue scores as time-dependent outcome variable.

**Models**	**Estimate**	***p***	**95% CI**
AUCg	0.00	0.79	−0.002 to 0.002
AUCi	0.00	0.92	−0.002 to 0.002
Night time cortisol (S5)	0.03	0.83	−0.25 to 0.31
DST ratio (S1/S6)	0.00	0.97	−0.06 to 0.06

*AUCg, area under the curve with respect to ground; AUCi, area under the curve with respect to increase; S1, sample upon awakening; S5, night-time sample; S6, sample upon awakening post-dexamethasone intake; DST, dexamethasone suppression test*.

### Effects of Interventions on Diurnal Cortisol Secretion

None of the interventions showed significant effects on AUCg, AUCi, night-time cortisol, and DST ratio (Table 3), except for a significant decrease in AUCi for the ECM intervention group over 52 weeks (β = −114.8, *p* = 0.007, 95% CI = −197.6, −31.9) (Table 3). No confounding effects of age, gender, and disease duration were observed on interventions and AUCg, S5, and DST.

**Table 3:** Effects of AT, ECM, and CBT on diurnal cortisol secretion (as dependent outcomes).

	**Estimate**	***p* value**	**95% CI**
**Model AUCg dependent outcome variable**			
AT	58.1	0.20	[−30.0, 146.3]
ECM	−17.5	0.67	[−105.1, 70.8]
CBT	45.5	0.28	[−36.5, 127.4]
**Model AUCi dependent outcome variable**			
AT	0.86	0.98	[−81.4, 83.1]
ECM	−114.8	*0.007*	[−197.7, −31.9]
CBT	−36.2	0.35	[−112.6, 40.2]
**Model Evening cortisol (S5) dependent outcome variable**			
AT	−0.22	0.45	[−0.79, 0.35]
ECM	0.01	0.96	[−0.55, 0.58]
CBT	−0.01	0.97	[−0.56, 0.54]
**Model DST ratio dependent outcome variable**			
AT	1.64	0.32	[−1.63, 4.91]
ECM	−0.97	0.54	[−4.13, 2.20]
CBT	2.69	0.08	[−0.34, 5.72]

*AT, aerobic training; ECM, energy conservation management; CBT, cognitive behavioral therapy; AUCg, area under the curve with respect to ground; AUCi, area under the curve with respect to increase; S1, sample upon awakening; S5, night-time sample; S6, sample upon awakening post-dexamethasone intake; DST, dexamethasone suppression test*.

*Results of linear mixed model*.

## Discussion

In this study, we focused on the role of HPA-axis function on primary fatigue in MS patients who participated in three identical randomized controlled trials that studied the effects of rehabilitation interventions (AT, ECM, and CBT) (13–15). Assessments of longitudinal salivary cortisol samples in fatigued MS patients showed no effect of diurnal cortisol parameters on CIS20R fatigue scores over the 1-year study period. Our results are in agreement with other studies finding no association of diurnal cortisol secretion parameters with MS-related fatigue (9, 10, 24, 25).

With respect to the specific intervention effects, only the ECM intervention led to a reduced dynamic daytime cortisol secretion. The ECM and AT rehabilitation strategies showed no significant reductions in MS-related fatigue compared to the control group, while the CBT intervention showed effective fatigue reduction on the short term (13–15). Noteworthy, the trial results in our study are based on the participants with available CAR included for this study, with pooling of the control groups as reference and therefore slightly vary with primary trial analyses of the TREFAMS studies (13–15). The biological mechanisms underlying the observed improvements have yet to be determined. Based on the stress managing effects of CBT and ECM therapy in MS patients, we hypothesized that HPA axis could have a mediating effect on MS-related fatigue (7, 9, 10, 16, 20). However, our data contests this hypothesis, since we neither observe any relation of salivary diurnal cortisol secretion with fatigue scores in MS patients nor in response to CBT. However, we observed a significant reduction in the dynamic increase in cortisol secretion upon awakening in the ECM trial group. No other studies so far have studied the effects of ECM on CAR. Possibly, attained ECM strategies to identify and, if necessary, adapt activities has resulted into a smaller cortisol surge upon awakening in the ECM intervention group.

Several studies report higher CAR values in MS patients compared to healthy controls (9, 10, 17). Whether the HPA-axis function was disturbed in our patient population is difficult to determine because we did not include healthy controls, and therefore, it is difficult to conclude whether a hyperactive CAR is present specifically in the fatigued patients of the TREFAMS cohort. Moreover, to our knowledge, no other studies have applied LC-MS/MS method for saliva cortisol measurements in MS patients; therefore, comparisons for diurnal cortisol secretion values between our study and previous reports are challenging. In contradiction with our results, others indicate faulty cortisol feedback mechanisms in 62% of MS patients assessed by the DST (12), whereas we only found a deviated response to DST in 6% of the participants. A possible explanation is the relative mild EDSS scores of the TREFAMS cohort, while in the previous study, EDSS scores of ~5 were assessed (12). It is possible that HPA-axis dysregulation is caused by autoimmune and neurodegenerative mechanisms upon disease progression, and because of the relative mild disease status of the TREFAMS cohort, we did not observe similar DST test results. This indicates that possible HPA-axis dysregulation observed by earlier studies is most likely a consequence of MS progression mechanisms. Overall, in this highly controlled longitudinal study, we found no evidence that diurnal cortisol secretion parameters are associated with primary fatigue in MS.

The major strength of our study is the longitudinal collection of salivary cortisol obtained from MS patients with primary fatigue by excluding MS patients with secondary fatigue (e.g., anemia, major depressive disorders, and sleep disorders). In addition, we selected homogenous groups of MS patients with primary fatigue by the application of additional exclusion criteria, such as relapse, pregnancy, and pharmacological and non-pharmacological treatments of fatigue. To our knowledge, this is the most extensive longitudinal study for MS-related fatigue to date, which allowed us to determine the potential longitudinal role of the HPA axis in MS-related fatigue (16).

Furthermore, we assessed salivary cortisol levels using LC-MS/MS approach, whereas most of the earlier studies have assessed serum and or urine cortisol levels using immunoassay (16, 21). In comparison with serum or urine cortisol levels, salivary cortisol levels reflect biological active and non-protein-bound cortisol levels that follow the circadian rhythm (21). In addition, saliva matrix is an ultrafiltrated matrix in comparison with serum (21). Despite the overall ease to use immunoassays for cortisol quantification, it is advised to perform prepurification before sample when using these assays (21). In addition, the use of LC-MS/MS for quantification of saliva, blood, and urine cortisol has major benefits over immunoassays (21). Especially in saliva samples, the cortisol concentrations are 10-fold lower than serum cortisol levels; therefore, a highly specific and sensitive assay is required (21). The LC-MS/MS assay used had an overall good intra-assay variation and sensitivity.

Several limitations of our study have to be acknowledged. In the TREFAMS-ACE trials, a total of 266 participants were randomized. However, a percentage of salivary assessments were not valid, could not be validly traced in the lab, or were not sent to the lab by the participants (13–15), although based on the similarities between the included and excluded participants for mean EDSS scores, age, and gender distribution, it is likely that similar results would have been observed especially due to the lack of any effects of the diurnal cortisol parameters on MS fatigue scores.

Owing to the nature of intervention, the participants, therapists, and MS nurses were not blinded (23). Therefore, contamination for example in the control group by applying self-researching for non-pharmacological treatments could have been possible. Nevertheless, cortisol lab analyses were performed by staff blinded to treatment allocation of the participants (16).

Furthermore, saliva was sampled by the patients themselves at home, and self-reported time of samplings was required. Therefore, compliance with instructions were essential, especially for the CAR because of its very characteristic curve within the first hour of awakening and its dependence on the awakening sampling time (26–28). Non-compliance by delayed sampling after awakening may explain why some patients showed a cortisol decrease (negative AUCi) after awakening. Others report a self-reported compliance rate of ~90%, whereas unaware monitored participants showed a concordance rate of ~71% (27–29). Another study reported, despite closely monitoring of participants, still a negative CAR in 15% of the participants (29). Thus, the small group with negative AUCi might biologically be of interest, and negative AUCi should be observed as an index of decrease and included in further analyses (19). Since compliance is an important parameter, although difficult to monitor by self-report, a combination of actigraph for registering sleep and awakening activities, and electronic sampling time of salivary tubes should be considered for future studies (27). Furthermore, it has been shown that AUCg and especially AUCi could be affected by situational factors (30). Therefore, to be able to obtain more reliable CAR for interpersonal comparisons, collection of saliva during six consecutive days is advised, instead of a 24-h sampling (30). However, this could be more challenging for MS patients with fatigue, resulting in a decrease in compliance, especially in a longitudinal setting.

Lastly, we did not exclude MS patients who were on immunomodulatory disease-modifying therapies at baseline. Including participants with MS-related fatigue that are not immune-modulating DMTs upon inclusion is challenging. Noteworthy, an earlier study included MS patients who were not using DMTs, and higher blood levels in MS patient with fatigue was observed (7). Interestingly, within the same study, no differences in cortisol blood levels were observed between MS patients with and without fatigue (7). This confirms our results and could indicate that immunomodulatory DMTs could affect the HPA axis and related corticosteroids differently, which could explain the observed adrenocorticotropic hormone levels in the earlier study (7).

In conclusion, we did not find any relation of changes in HPA-axis diurnal cortisol secretion and changes in MS-related fatigue. Furthermore, most HPA-axis parameters were not influenced by the type of intervention (CBT, ECM, or AT), with the exception of ECM reducing AUCi of the CAR. Our results indicate that MS-related fatigue cannot be attributed to HPA-axis diurnal cortisol secretion and is likely caused by other disease mechanisms.

## Data Availability Statement

The datasets generated for this study are available on request to the corresponding author.

## Ethics Statement

The studies involving human participants were reviewed and approved by VU University Medical Center. The patients/participants provided their written informed consent to participate in this study.

## Author Contributions

HB, VG, CT, and AM: study concept and design. AM, IB, and JD: data acquisition and analysis. AM, HB, VG, and CT: drafting significant portions of the manuscript or figures. Statistical analyses were done under supervision by JT. All authors critically reviewed and approved the final manuscript.

### Conflict of Interest

The authors declare that the research was conducted in the absence of any commercial or financial relationships that could be construed as a potential conflict of interest. The reviewer ET and handling editor declared their shared affiliation at the time of the review.

## References

[1] Nagaraj K Taly AB Gupta A Prasad C Christopher R. Prevalence of fatigue in patients with multiple sclerosis and its effect on the quality of life. J Neurosci. Rural Pract. (2013) 4:278–82. 10.4103/0976-3147.118774 24250159 PMC3821412

[2] Weiland TJ Jelinek GA Marck CH Hadgkiss EJ van der Meer DM Pereira NG. Clinically significant fatigue: prevalence and associated factors in an international sample of adults with multiple sclerosis recruited via the internet. PLoS ONE. (2015) 10:e0115541. 10.1371/journal.pone.0115541 25692993 PMC4333355

[3] Kos D Kerckhofs E Nagels G D'hooghe MB Ilsbroukx S. Origin of fatigue in multiple sclerosis: review of the literature. Neurorehabil. Neural Repair. (2008) 22:91–100. 10.1177/1545968306298934 17409388

[4] Braley TJ Chervin RD. Fatigue in multiple sclerosis: mechanisms, evaluation, and treatment. Sleep. (2010) 33:1061–7. 10.1093/sleep/33.8.1061 20815187 PMC2910465

[5] Smith MM Arnett PA. Factors related to employment status changes in individuals with multiple sclerosis. Mult. Scler. (2005) 11:602–9. 10.1191/1352458505ms1204oa 16193900

[6] Dantzer R Heijnen CJ Kavelaars A Laye S Capuron L. The neuroimmune basis of fatigue. Trends Neurosci. (2014) 37:39–46. 10.1016/j.tins.2013.10.003 24239063 PMC3889707

[7] Gottschalk M Kümpfel T Flachenecker P Uhr M Trenkwalder C Holsboer F. Fatigue and regulation of the hypothalamo-pituitary-adrenal axis in multiple sclerosis. Arch Neurol. (2005) 62:277–80. 10.1001/archneur.62.2.277 15710856

[8] Chaudhuri A Behan PO. Fatigue in neurological disorders. Lancet. (2004) 363:978–88. 10.1016/S0140-6736(04)15794-2 15043967

[9] Powell DJH Liossi C Moss-Morris R Schlotz W. Unstimulated cortisol secretory activity in everyday life and its relationship with fatigue and chronic fatigue syndrome: a systematic review and subset meta-analysis. Psychoneuroendocrinology. (2013) 38:2405–22. 10.1016/j.psyneuen.2013.07.004 23916911

[10] Powell DJH Moss-Morris R Liossi C Schlotz W. Circadian cortisol and fatigue severity in relapsing-remitting multiple sclerosis. Psychoneuroendocrinology. (2015) 56:120–31. 10.1016/j.psyneuen.2015.03.010 25817406

[11] Fries E Dettenborn L Kirschbaum C. The cortisol awakening response (CAR): facts and future directions. Int J Psychophysiol. (2009) 72:67–73. 10.1016/j.ijpsycho.2008.03.014 18854200

[12] Ysrraelit MC Gaitan MI Lopez AS Correale J. Impaired hypothalamic-pituitary-adrenal axis activity in patients with multiple sclerosis. Neurology. 71:1948–54. 10.1212/01.wnl.0000336918.32695.6b 19064876

[13] van den Akker LE Beckerman H Collette EH Twisk JW Bleijenberg G Dekker J. Cognitive behavioral therapy positively affects fatigue in patients with multiple sclerosis: results of a randomized controlled trial. Mult Scler J. (2017) 23:1542–53. 10.1177/1352458517709361 28528567

[14] Blikman LJ van Meeteren J Twisk JW de Laat FA de Groot V Beckerman H. Effectiveness of energy conservation management on fatigue and participation in multiple sclerosis: a randomized controlled trial. Mult Scler J. (2017) 23:1527–41. 10.1177/1352458517702751 28528565

[15] Heine M Verschuren O Hoogervorst EL van Munster E Hacking HG Visser-Meily A. Does aerobic training alleviate fatigue and improve societal participation in patients with multiple sclerosis? A randomized controlled trial. Mult Scler J. (2017) 23:1517–26. 10.1177/1352458517696596 28528566 PMC5624301

[16] Beckerman H Sanches S Blikman L Heine M Malekzadeh A Treating fatigue in multiple sclerosis: aerobic training, cognitive behavioural therapy, energy conservation management: The TREFAMS-ACE study design (#5). Trials. (2013) 14:250. 23938046 10.1186/1745-6215-14-250 PMC3751829

[17] Gold SM Krüger S Ziegler KJ Krieger T Schulz KH Otte C. Endocrine and immune substrates of depressive symptoms and fatigue in multiple sclerosis patients with comorbid major depression. J Neurol Neurosurg Psychiatry. (2011) 82:814–8. 10.1136/jnnp.2010.230029 21296901

[18] Stranahan AM Lee K Mattson MP. Central mechanisms of HPA axis regulation by voluntary exercise. Neuro Mol Med. (2008) 10:118–27. 10.1007/s12017-008-8027-0 18273712 PMC3010733

[19] Pruessner JC Kirschbaum C Meinlschmid G Hellhammer DH. Two formulas for computation of the area under the curve represent measures of total hormone concentration versus time-dependent change. Psychoneuroendocrinology. (2003) 28:916–31. 10.1016/S0306-4530(02)00108-7 12892658

[20] Heesen C Gold SM Raji A Wiedemann K Schulz KH. Cognitive impairment correlates with hypothalamo-pituitary-adrenal axis dysregulation in multiple sclerosis. Psychoneuroendocrinology. (2002) 27:505–17. 10.1016/S0306-4530(01)00071-3 11912002

[21] Turpeinen U Hämäläinen E. Determination of cortisol in serum, saliva and urine. Best Pract Res Clin Endocrinol Metab. (2013) 27:795–801. 10.1016/j.beem.2013.10.008 24275191

[22] Vreeburg SA Hoogendijk WJ DeRijk RH van Dyck R Smit JH Zitman FG. Salivary cortisol levels and the 2-year course of depressive and anxiety disorders. Psychoneuroendocrinology. (2013) 38:1494–502. 10.1016/j.psyneuen.2012.12.017 23313277

[23] Andrews K The limitations of randomized controlled trials in rehabilitation research. Clin Rehabil. (1991) 5:5–8. 10.1177/026921559100500102

[24] Akcali A Zengin F Aksoy SN Zengin O. Fatigue in multiple sclerosis: is it related to cytokines and hypothalamic-pituitary-adrenal axis? Mult Scler Relat Disord. (2017) 15:37–41. 10.1016/j.jns.2017.08.674 28641771

[25] Heesen C Nawrath L Reich C Bauer N Schulz KH Gold SM. Fatigue in multiple sclerosis: An example of cytokine mediated sickness behaviour? J Neurol Neurosurg Psychiatry. (2006) 77:34–9. 10.1136/jnnp.2005.065805 16361589 PMC2117393

[26] Kudielka BM Kirschbaum C Awakening cortisol responses are influenced by health status and awakening time but not by menstrual cycle phase. Psychoneuroendocrinology. (2003) 28:35–47. 10.1016/S0306-4530(02)00008-2 12445835

[27] Kudielka BM Broderick JE Kirschbaum C. Compliance with saliva sampling protocols: electronic monitoring reveals invalid cortisol daytime profiles in noncompliant subjects. Psychosom Med. (2003) 65:313–9. 10.1097/01.PSY.0000058374.50240.BF 12652000

[28] Broderick JE Arnold D Kudielka BM Kirschbaum C. Salivary cortisol sampling compliance: comparison of patients and healthy volunteers. Psychoneuroendocrinology. (2004) 29:636–50. 10.1016/S0306-4530(03)00093-3 15041086

[29] Dockray S Bhattacharyya MR Molloy GJ Steptoe A. The cortisol awakening response in relation to objective and subjective measures of waking in the morning. Psychoneuroendocrinology. (2008) 33:77–82. 10.1016/j.psyneuen.2007.10.001 17996380

[30] Hellhammer J Fries E Schweisthal OW Schlotz W Stone AA Hagemann D. Several daily measurements are necessary to reliably assess the cortisol rise after awakening: State- and trait components. Psychoneuroendocrinology. (2007) 32:80–6. 10.1016/j.psyneuen.2006.10.005 17127010

